# Comparing adults with severe SARS-CoV-2 or influenza infection: South Africa, 2016–2021

**DOI:** 10.4102/sajid.v39i1.574

**Published:** 2024-07-26

**Authors:** Fiona Els, Jackie Kleynhans, Nicole Wolter, Mignon du Plessis, Fahima Moosa, Stefano Tempia, Mvuyo Makhasi, Jeremy Nel, Halima Dawood, Susan Meiring, Anne von Gottberg, Cheryl Cohen, Sibongile Walaza

**Affiliations:** 1 Centre for Respiratory Diseases and Meningitis, National Institute for Communicable Diseases (NICD) of the National Health Laboratory Service (NHLS), Johannesburg, South Africa; 2 South African Field Epidemiology Training Programme (SAFETP), Division of Public Health, Surveillance and Response (DPHSR), National Institute for Communicable Diseases (NICD) of the National Health Laboratory Service (NHLS), Johannesburg, South Africa; 3 School of Health Systems and Public Health, Faculty of Health Sciences, University of Pretoria, Pretoria, South Africa; 4 School of Public Health, Faculty of Health Sciences, University of the Witwatersrand, Johannesburg, South Africa; 5 School of Pathology, Faculty of Health Sciences, University of the Witwatersrand, Johannesburg, South Africa; 6 Department of Medicine, Faculty of Health Sciences, University of the Witwatersrand, Johannesburg, South Africa; 7 Department of Medicine, Greys Hospital, Pietermaritzburg and Centre for the Aids programme of research in South Africa, University of KwaZulu-Natal, Pietermaritzburg, South Africa; 8 Division of Public Health Surveillance and Response, National Institute for Communicable Diseases of the National Health Laboratory Service, Johannesburg, South Africa

**Keywords:** COVID-19, pneumonia surveillance, risk factors, severe respiratory illness, HIV, pre-pandemic

## Abstract

**Background:**

Comparisons of the characteristics of individuals hospitalised with severe acute respiratory syndrome coronavirus 2 (SARS-CoV-2) or seasonal influenza in low-to middle-income countries with high human immunodeficiency virus (HIV) prevalence are limited.

**Objectives:**

Determine the epidemiological differences with those hospitalised with influenza or SARS-CoV-2 infection.

**Method:**

We investigated hospitalised individuals ≥18 years of age testing positive for seasonal influenza (2016–2019) or SARS-CoV-2 (2020–2021). We used random effects multivariable logistic regression, controlling for clustering by site, to evaluate differences among adults hospitalised with influenza or SARS-CoV-2 infection.

**Results:**

Compared to individuals with influenza, individuals with SARS-CoV-2 infection were more likely to be diabetic (adjusted odds ratio [aOR]: 1.70, 95% confidence interval [CI]: 1.11–2.61) or die in hospital (aOR: 2.57, 95% CI: 1.61–4.12). Additionally, those with SARS-CoV-2 infection were less likely to be living with HIV (not immunosuppressed) (aOR: 0.50, 95% CI: 0.34–0.73) or living with HIV (immunosuppressed) (aOR: 0.27, 95% CI: 0.18–0.39) compared to not living with HIV and less likely to be asthmatic (aOR: 0.21, 95% CI: 0.13–0.33) rather than those living with influenza.

**Conclusion:**

Individuals hospitalised with SARS-CoV-2 had different characteristics to individuals hospitalised with influenza before the coronavirus disease 2019 (COVID-19) pandemic. Risk factors should be considered in health management especially as we move into an era of co-circulation of SARS-CoV-2 and influenza pathogens.

**Contribution:**

Identifying groups at high risk of severe disease could help to better monitor, prevent and control SARS-CoV-2 or influenza severe disease.

## Background

Severe acute respiratory syndrome coronavirus 2 (SARS-CoV-2) and influenza have both caused pandemics, with influenza also causing seasonal epidemics and the post-pandemic epidemiology of SARS-CoV-2 still being unknown. In South Africa, by 31 December 2021, 3 485 286 laboratory-confirmed cases of SARS-CoV-2 had been reported, with 91 648 deaths.^1^ Groups at a higher risk of severe disease and mortality because of coronavirus disease 2019 (COVID-19) include those aged older than 60 years of age, males, and individuals with hypertension, diabetes, chronic cardiac disease, obesity, living with HIV, chronic renal disease, malignancy, and tuberculosis.^2,3,4^

Globally, influenza virus infection causes substantial mortality and morbidity among adults aged 65 years and older and children under 5 years of age.^5^ In South Africa, seasonal influenza infections cause approximately 56 000 hospitalisations and 11 000 deaths annually.^6,7^ The severity of influenza depends on several factors, including the infecting subtypes, natural and vaccine-induced immunity, age and the health condition of the population.^8^ Annual epidemics pose a risk of severe disease to all age groups, but the highest risk of severe disease lies with children younger than 5 years, adults older than 65 years, and any person with an underlying illness such as asthma, diabetes, chronic heart and lung disease, obesity, and human immunodeficiency virus (HIV) infection.^5^ South Africa has a high HIV prevalence, with 7.3 million people over the age of 15 years living with HIV in 2021.^9^ People living with HIV are at higher risk of influenza-associated pneumonia and hospitalisation.^10^

While respiratory disease caused by seasonal influenza is well described in South Africa,^5,11^ SARS-CoV-2 is a novel pathogen and comparing disease epidemiology to that of seasonal influenza may provide useful information.^12^ To control the spread of SARS-CoV-2, the South African government, declared a national state of disaster on 15 March 2020 after the first cases had been confirmed on 05 March 2020 when all domestic and international travel were restricted, and schools were closed. The initial lockdown (Alert level 5) started on 27 March 2020, after only 51 cases were confirmed, was for 3 weeks and extended for another 3 weeks.^13^ Over the next 2 years, there was a step-wise relaxation of restrictions, beginning 01 May 2020,^14^ the final state of disaster being declared on 05 April 2022.^15^

A few developed countries have compared SARS-CoV-2 infection to those with influenza infection.^16,17,18^ However, studies comparing the demographic and clinical differences between patients hospitalised with SARS-CoV-2 or seasonal influenza in a low-to-middle income (LMIC) setting with high HIV prevalence are limited. Additionally, SARS-CoV-2 is currently co-circulating with influenza. Identifying groups at high risk of severe disease could help to guide prevention and treatment strategies, such as vaccines or clinical management. We aimed to determine the demographic and clinical differences between adult individuals (≥ 18 years) hospitalised with SARS-CoV-2 during the first 2 years of the COVID-19 pandemic and seasonal influenza infection prior to the pandemic.

## Research methods and design

### Study design

This was a cross-sectional study using data from the pneumonia surveillance programme for the period 2016–2021, including SARS-CoV-2 data (March 2020 until December 2021) and influenza data (for January 2016 until December 2019).

### Study setting: Pneumonia surveillance programme

Syndromic surveillance for severe respiratory illness is performed at sentinel sites located in 5 out of 9 South African provinces and was established in 2009 by the Centre for Respiratory Diseases and Meningitis of the National Institute for Communicable Diseases (NICD).^19^

### Participants

Hospitalised patients meeting the case definition were enrolled in the surveillance programme as previously described.^20,21^ The case definition for severe respiratory illness in adults in the pre-pandemic era (2009–February 2020) was any person hospitalised with a physician-diagnosed lower respiratory tract infection and fever ≥ 38 °C or history of fever and cough. The case definition changed after the emergence of SARS-CoV-2 in South Africa to hospitalised individuals with physician-diagnosed lower respiratory tract infection or if COVID-19 was suspected by physician (March 2020–2021).^20^ Consenting hospitalised patients of all ages who met the case definition were enrolled; however, this analysis was restricted to adults aged ≥ 18 years of age.

### Laboratory procedures

Surveillance officers collected demographic and clinical information from structured interviews and hospital records. Nasopharyngeal swabs were collected in universal transport medium and were routinely tested for influenza virus, respiratory syncytial virus (RSV) and *Bordetella pertussis.* In 2020, testing for SARS-CoV-2 was added. During 2016–2019, nasopharyngeal swabs (Copan Italia, Brescia, Italy) were collected and transported within 72 h in universal transport medium at 4 °C – 8 °C to the reference laboratory at the NICD. The nasopharyngeal swabs were tested for influenza and human respiratory syncytial virus (HRSV) using a Flu and HRSV commercial multiplex real-time reverse transcription polymerase chain reaction kit (Fast-Track Diagnostics, Luxembourg City, Luxembourg). From 01 April 2020 to 28 February 2021, nasopharyngeal swabs were additionally tested for SARS-CoV-2 using the TIB MOLBIOL E gene assay (Roche Diagnostics, Mannheim, Germany).^22^ From 01 March 2021, nasopharyngeal swabs were tested using the Allplex^™^ SARS-CoV-2/Flu A/Flu B/RSV kit (Seegene, Seoul, South Korea) as previously described.^20^

### Variables and statistical analysis

#### Quantitative variables

Human immunodeficiency virus status was categorised in accordance with the World Health Organization (WHO) staging guidelines for the African Region^23^: people not living with HIV (negative HIV test at current admission or within 6 months of admission), people living with HIV not immunosuppressed (positive HIV test, low viral load [< 400 copies/mL] or normal CD4 count [200–1500 cells per cubic millimetre of blood]), people living with HIV immunosuppressed (positive HIV test, low CD4 count [≤ 200 cells/mm^3^ of blood] irrespective of viral load, or high viral load [≥ 400 copies/mL]), people living with HIV immunosuppression unknown (positive HIV test, viral load and CD4 count unknown), HIV status unknown. Body mass index (BMI) was classified as underweight < 18.5 kg/m^2^, normal 18.5 kg/m^2^–24.9 kg/m^2^, overweight 25 kg/m^2^–30 kg/m^2^ or obese > 30 kg/m^2^.^24^ Mechanical ventilation includes both invasive and non-invasive mechanical ventilation. Mortality in this study was defined as in-hospital death and does not include palliative discharges. We expected that non-complicated pneumonia cases will be discharged within an average of 3–5 days, and any hospitalisation more than 5 days was deemed complicated. Age data were categorised into four groups: 18–24 years, 25–44 years, 45–65 years, ≥ 65 years to determine age groups most associated with SARS-CoV-2 or influenza infection, and to preserve power within categories.

#### Statistical analysis

**FIGURE 1:**
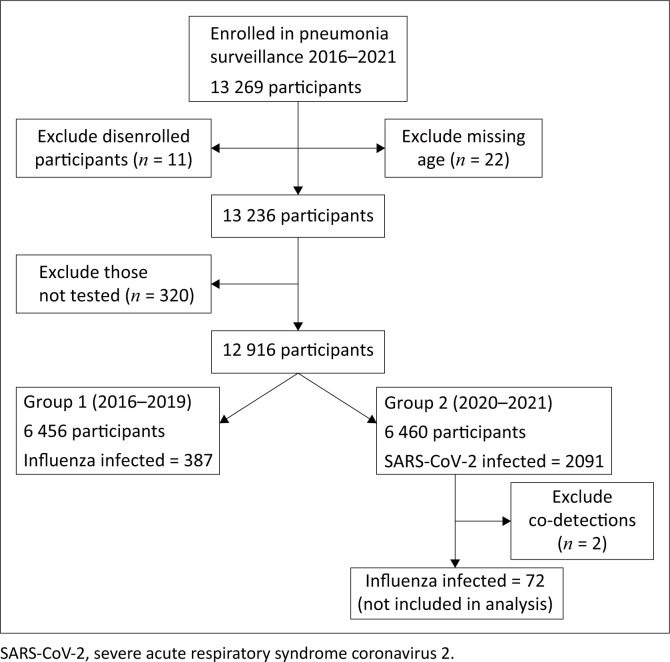
Study flow diagram for inclusion of individuals aged ≥ 18 years enrolled in the pneumonia surveillance programme between 2016 and 2021.

During 2020–2021, there were four waves of SARS-CoV-2 infections in South Africa, and this was compared with four seasons of influenza infections (2016–2019). Data analysis was performed to compare demographic and clinical characteristics of adult patients hospitalised with SARS-CoV-2 or influenza infection. We characterised variables according to period (2016–2019 and 2020–2021), and cross tabulated the variables with the outcome (influenza positive or SARS-CoV-2 positive). Any influenza and SARS-CoV-2 co-detections and missing age data were excluded. Missing data were coded in an ‘unknown’ category. In addition, we performed a chi-squared test to determine statistically significant (*p* ≤ 0.05) differences between the two periods under consideration (Group 1 and Group 2, Figure 1).

We used logistic regression to determine the association between demographic and clinical characteristics of patients hospitalised with severe respiratory illness with laboratory-confirmed SARS-CoV-2 or influenza infection, with random effects to account for clustering by site. The outcome of interest was SARS-CoV-2 (coded as 1) or influenza infection (coded as 0), and the analysis was performed in patients with laboratory-confirmed SARS-CoV-2 in 2020–2021, compared with those with laboratory-confirmed influenza during 2016–2019. Influenza infections in 2020–2021 were excluded as these may not have reflected a typical seasonal influenza.^25^ We tested all variables on univariate analysis (Table 2) and evaluated any characteristics with *p* < 0.2 in the multivariable analysis. Manual backward step-wise elimination was done and verified with manual forward selection, resulting in the same model, retaining any variables with *p* < 0.05 in the final model (Table 2). Sensitivity analysis, using the same regression approach was performed to determine the effect of an unknown immunosuppression status with people living with HIV. This was carried out by coding unknown immunosuppression status into the immunosuppressed category, and then also coding the unknown immunosuppression status into the not immunosuppressed category and assessing the differences in ratios and *p*-values. Data were analysed using Stata version 16 (StataCorp LLC, College Station, Texas, United States, 2019).

### Ethical considerations

The pneumonia surveillance protocol was approved by the University of the Witwatersrand Human Research Ethics Committee (HREC), reference M140824; and this study protocol was approved by the University of Pretoria HREC, reference 696/2021.

## Results

Overall, 6456 and 6458 adults were enrolled during 2016–2019 and 2020–2021 in the pneumonia surveillance programme, respectively (Figure 1). Among all enrolled individuals, 54.2% were female (6999/12 914), 87.1% were of black people (11 249/12 914) and 43.6% were aged between 25 and 44 years (5636/12 914) (Table 1).

**TABLE 1:** Characteristics of hospitalised patients aged ≥ 18 years enrolled in the pneumonia surveillance programme, South Africa, 2016–2021.

Characteristic	Overall patients enrolled				*p*
	2016–2019 (*n* = 6456)		2020–2021 (*n* = 6458)		
	*n*	%	*n*	%
**Site (province)**					
Edendale (KZN)	1497	23	1507	23	**< 0.001**
HJH-RMMCH (GP)	1993	31	1908	30	
Klerksdorp-Tshepong (NW)	1853	29	1292	20	
Mapulaneng-Matikwana (MP)	558	9	813	13	
Mitchells Plain (WC)	555	9	938	15	
**Age group (years)**					
18–24	461	7	304	5	**< 0.001**
25–44	3304	51	2332	36	
45–64	1954	30	2438	38	
≥ 65	737	11	1384	21	
**Sex**					
Male	3024	47	2891	45	**0.018**
Female	3432	53	3567	55	
**Race**					
Black people	5827	90	5422	84	**< 0.001**
Other	629	10	1036	16	
**HIV infection**					
People not living with HIV	2155	33	3593	56	**< 0.001**
People living with HIV not immunosuppressed	852	13	747	12	
People living with HIV immunosuppressed	1875	29	1081	17	
People living with HIV immunosuppression unknown	1507	23	666	10	
HIV status unknown	67	1	371	6	
**Underlying conditions**					
BMI category					
Underweight	1196	18	469	7	**< 0.001**
Normal	2730	42	1829	27	
Overweight	1142	18	1232	18	
Obese	896	14	1642	24	
Unknown	501	8	1594	24	
Asthma					
No	6121	95	6119	95	**0.003**
Yes	332	5	320	5	
Unknown	3	0	19	0	
Diabetes					
No	6102	95	5496	85	**< 0.001**
Yes	351	5	945	15	
Unknown	3	0	17	0	
**Other underlying conditions** †					
No	5635	87	5103	79	**< 0.001**
Yes	757	12	1284	20	
Unknown	64	1	71	1	
**Disease severity**					
Oxygen therapy					
No	3601	56	2007	31	**< 0.001**
Yes	2844	44	4410	68	
Unknown	11	0	41	1	
Mechanical ventilation					
No	6415	99	6339	98	**< 0.001**
Yes	27	0	78	1	
Unknown	14	0	41	1	
Duration of hospitalisation ≥ 5 days					
No	2397	37	2523	39	**0.023**
Yes	4059	63	3935	61	
ICU admission					
No	6399	99	6355	98	**< 0.001**
Yes	43	1	62	1	
Unknown	14	0	41	1	
Outcome					
Survived	5969	92	5562	86	**< 0.001**
Died	482	7	832	13	
Unknown	5	0	64	1	

Note: Boldface values are statistically significant and indicate *p* < 0.05 as calculated using a Chi-square test. People living with HIV not immunosuppressed – positive HIV test, low viral load (< 400 copies/mL) or normal CD4 count (200 cells – 1500 cells per cubic millimetre of blood), people living with HIV immunosuppressed – positive HIV test, high viral load (> 400 copies/mL) or low CD4 count (< 200 cells per cubic millimetre of blood), people living with HIV immunosuppression unknown – positive HIV test, viral load or CD4 count unknown, HIV status unknown – no data for HIV, BMI category defined as underweight < 18.5 kg, normal 18.5 kg – 24.9 kg, overweight 25 kg – 30 kg, or obese > 30 kg.

ICU, intensive care unit; HIV, human immunodeficiency virus; BMI, body mass index; HJH-RMMCH, Helen Joseph Hospital and Rahima Moosa Mother and Child Hospital complex; KZN, KwaZulu-Natal; GP, Gauteng; NW, North West; MP, Mpumalanga; WC, Western Cape.

† , Other underlying conditions included any of the following: anaemia, asplenia, burns, chronic heart disease (valvular heart disease, coronary heart disease, or heart failure excluding hypertension), emphysema, immunocompromising conditions excluding HIV infection (organ transplant, immunosuppressive therapy, immunoglobulin deficiency, liver disease (cirrhosis or liver failure), malignancy, autoimmune disease), neurological disease (spinal cord injury, neuromuscular conditions), obesity, other chronic lung diseases, pregnancy, renal disease (nephrotic syndrome, chronic renal failure), seizures, stroke.

**FIGURE 2:**
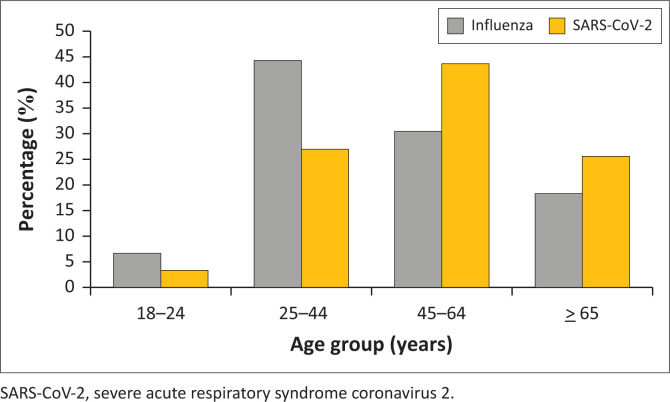
Distribution of influenza (*n* = 387) and SARS-CoV-2 (*n* = 2091) cases by age group of individuals aged ≥ 18 years enrolled in the pneumonia surveillance programme between 2016 and 2021.

Of those enrolled during 2016–2019, 387/6465 (6.0%) had influenza. Of these, 242/387 (62.0%) were female and 339/387 (87.6%) were of black people. Among those hospitalised with influenza, 25/387 (6.5%) died in hospital, 172/387 (44.4%) were 25–44 years of age (Table 2). Among those hospitalised with SARS-CoV-2, 567/2091 (27.1%) were 25–44 years of age (Figure 2).

**TABLE 2:** Logistic regression analysis of epidemiological and clinical differences between patients aged ≥ 18 years with laboratory-confirmed influenza or SARS-CoV-2 infection, pneumonia surveillance programme, South Africa, 2016–2021 (*N* = 2478).

Characteristic	Influenza - Positive (*n* = 387)		SARS-CoV-2 - Positive (*n* = 2091)		Univariate analysis			Multivariable analysis		
	*n*	%	*n*	%	OR	95% CI	*p*	aOR	95% CI	*p*
**Site (province)**										
Edendale (KZN)	77	20	499	24	**Reference**	**Reference**	-	-	-	-
HJH-RMMCH (GP)	112	29	544	26	0.75	0.55–1.03	0.072	-	-	-
Klerksdorp-Tshepong (NW)	106	27	491	23	0.71	0.52–0.98	**0.039**	-	-	-
Mapulaneng-Matikwana (MP)	57	15	196	9	0.53	0.36–0.78	**0.001**	-	-	-
Mitchells Plain (WC)	35	9	361	17	1.59	1.04–2.43	**0.031**	-	-	-
**Age group (years)**										
18–24	26	7	67	3	**Reference**	**Reference**		**Reference**	**Reference**	
25–44	172	44	567	27	1.25	0.77–2.04	0.365	0.80	0.45–1.41	0.437
45–64	118	30	917	44	2.93	1.79–4.81	**< 0.001**	1.06	0.59–1.89	0.853
≥ 65	71	18	540	26	2.94	1.75–4.95	**< 0.001**	0.58	0.31–1.08	0.088
**Sex**										
Male	145	37	795	38	Reference	Reference	-	-	-	-
Female	242	63	1296	62	0.99	0.79–1.24	0.926	-	-	-
**Race**										
Black people	339	88	80	80	0.65	0.45–0.93	**0.017**	-	-	-
Other	48	12	20	20	**Reference**	**Reference**	-	-	-	-
**HIV infection**										
People not living with HIV	156	40	1442	69	**Reference**	**Reference**	-	**Reference**	**Reference**	-
People living with HIV not immunosuppressed	56	14	213	10	0.42	0.30–0.58	**< 0.001**	0.50	0.34–0.73	**< 0.001**
People living with HIV immunosuppressed	71	18	140	7	0.22	0.16–0.31	**< 0.001**	0.27	0.18–0.39	**< 0.001**
People living with HIV immunosuppression unknown	100	26	144	7	0.16	0.12–0.22	**< 0.001**	0.18	0.13–0.26	**< 0.001**
HIV status unknown	4	1	152	7	4.32	1.57–11.89	**0.005**	4.34	1.52–12.41	**0.006**
**Underlying conditions**										
BMI category										
Underweight	58	15	50	2	0.35	0.23–0.54	**< 0 .001**	0.40	0.25–0.66	-
Normal	146	38	362	17	**Reference**	**Reference**	-	**Reference**	**Reference**	**< 0.001**
Overweight	74	19	465	22	2.38	1.74–3.27	**< 0.001**	1.76	1.25–2.49	**0.001**
Obese	80	21	771	37	3.72	2.74–5.05	**< 0.001**	2.57	1.84–3.58	**< 0.001**
Unknown	29	7	443	21	7.81	5.02–12.16	**< 0.001**	4.45	2.78–7.12	**< 0.001**
Asthma										
No	347	90	2014	96	**Reference**	**Reference**	-	**Reference**	**Reference**	-
Yes	40	10	73	3	0.29	0.19–0.43	**< 0 .001**	0.21	0.13–0.33	**< 0.001**
Unknown	0	0	4	0	N/A	N/A	-	-	-	-
Diabetes										
No	356	92	1641	78	**Reference**	**Reference**	-	**Reference**	**Reference**	-
Yes	31	8	446	21	2.96	2.01–4.34	**< 0.001**	1.70	1.11–2.61	**0.014**
Unknown	0	0	4	0	N/A	N/A	-	-	-	-
**Other underlying illness** †										
No	315	81	1620	77	**Reference**	**Reference**	-	-	-	-
Yes	71	18	460	22	1.24	0.94–1.65	0.128	-	-	-
Unknown	1	0	11	1	2.16	0.28–16.86	0.463	-	-	-
**Disease severity**										
Oxygen therapy										
No	203	52	460	22	**Reference**	**Reference**	-	-	-	-
Yes	184	48	1619	77	3.75	2.97–4.73	**< 0.001**	3.01	2.30–3.93	**< 0.001**
Unknown	0	0	12	1	N/A	N/A	-	-	-	-
Mechanical ventilation										
No	381	98	2032	97	**Reference**	**Reference**	-	-	-	-
Yes	5	1	47	2	1.78	0.70–4.52	0.225	-	-	-
Unknown	1	0	12	1	2.45	0.32–19.02	0.391	-	-	-
Duration of hospitalisation ≥ 5 days										
No	177	46	932	45	**Reference**	**Reference**	-	-	-	-
Yes	210	54	1159	55	1.05	0.84–1.32	0.653	-	-	-
ICU admission										
No	378	98	2033	97	**Reference**	**Reference**	-	-	-	-
Yes	8	2	46	2	1.10	0.51–2.36	0.808	-	-	-
Unknown	1	0	12	1	2.43	0.31–18.87	0.395	-	-	-
Outcome										
Survived	362	94	1709	82	**Reference**	**Reference**	-	**Reference**	**Reference**	-
Died	25	6	356	17	4.57	2.96–7.05	**< 0.001**	2.57	1.61–4.13	**< 0.001**
Unknown	0	0	26	1	N/A	N/A	-	-	-	-

Note: Boldface values are statistically significant and indicate *p* < 0.05 as calculated using a Chi-squared test. People living with HIV not immunosuppressed- positive HIV test, low viral load (< 400 copies/mL) or normal CD4 count (200 cells – 1500 cells per cubic millimetre of blood), people living with HIV immunosuppressed – positive HIV test, high viral load (> 400 copies/mL) or low CD4 count (< 200 cells per cubic millimetre of blood), people living with HIV immunosuppression unknown – positive HIV test, viral load or CD4 count unknown, HIV status unknown- no data for HIV, BMI category defined as underweight < 18.5 kg, normal 18.5 kg – 24.9 kg, overweight 25 kg – 30 kg, or obese > 30 kg.

SARS-CoV-2, severe acute respiratory syndrome coronavirus; OR, odds ratio; aOR, adjusted odds ratio; CI, confidence interval; N/A, omitted; ICU, intensive care unit; HIV, human immunodeficiency virus; BMI, body mass index; HJH-RMMCH, Helen Joseph Hospital and Rahima Moosa Mother and Child Hospital complex; KZN, KwaZulu-Natal; GP, Gauteng; NW, North West; MP, Mpumalanga; WC, Western Cape.

† , Other underlying conditions included any of the following: anaemia, asplenia, burns, chronic heart disease (valvular heart disease, coronary heart disease, or heart failure excluding hypertension), emphysema, immunocompromising conditions excluding HIV infection (organ transplant, immunosuppressive therapy, immunoglobulin deficiency, liver disease (cirrhosis or liver failure), malignancy, autoimmune disease), neurological disease (spinal cord injury, neuromuscular conditions), obesity, other chronic lung diseases, pregnancy, renal disease (nephrotic syndrome, chronic renal failure), seizures, stroke.

Two patients with co-detections of influenza and SARS-CoV-2 were excluded from further analysis. Among patients enrolled during 2020–2021, 2091/6458 (32.4%) tested positive for SARS-CoV-2 (Figure 1). During this period, 72 individuals tested positive for influenza, and were excluded from the analysis. Among those infected with SARS-CoV-2, 1296/2091 (62.0%) were female and 1678/2091 (80.2%) were of black people (Table 2). Among those hospitalised with SARS-CoV-2, 356/2091 (17.0%) died in hospital.

On multivariable analysis, after adjusting for site, compared with those with a normal BMI, overweight (aOR: 1.76, 95% CI: 1.25–2.49) or obese (aOR: 2.57, 95% CI: 1.84–3.58) individuals were more likely to have SARS-CoV-2 rather than influenza. In addition, compared to individuals without diabetes, those with diabetes were more likely to have SARS-CoV-2 (aOR: 1.70, 95% CI: 1.11–2.61) rather than influenza. Compared to those who survived, those who died in hospital were more likely to have SARS-CoV-2 (aOR: 2.57, 95% CI: 1.61–4.13) rather than influenza. In contrast, compared to people not living with HIV, people living with HIV and immunosuppressed (aOR: 0.27, 95% CI: 0.18–0.39) and people living with HIV and not immunosuppressed (aOR: 0.50, 95% CI: 0.34–0.73) were more likely to have influenza rather than SARS-CoV-2. Compared to those without asthma, individuals with asthma (aOR: 0.21, 95% CI: 0.13–0.33) were more likely to have influenza, rather than SARS-CoV-2. Finally, compared to those with a normal BMI, those who were underweight (aOR: 0.40, 95% CI: 0.25–0.66) were more likely to have influenza, rather than SARS-CoV-2 (Table 2).

In the sensitivity analysis (Table 3), when classifying all people living with HIV unknown immunosuppression as either immunosuppressed or not immune supressed, individuals hospitalised with influenza were still more likely to be people living with HIV immunosuppressed (OR: 0.15, 95% CI: 0.11–0.19) or not-immunosuppressed (OR: 0.26, 95% CI: 0.20–0.34), compared to those with SARS-CoV-2.

**TABLE 3:** Sensitivity analysis for patients aged ≥ 18 years with laboratory-confirmed influenza or SARS-CoV-2 infection, pneumonia surveillance programme, South Africa, 2016–2021.

HIV classification	Influenza (*n* = 387)		SARS-CoV-2 (*n* = 2091)		Univariate analysis		
	*n*	%	*n*	%	OR	95% CI	*p*
**HIV-positive original**							
People not living with HIV	156	40	1442	69	**Reference**	**Reference**	-
People living with HIV not immunosuppressed	56	14	213	10	0.42	0.30–0.59	< 0.001
People living with HIV immunosuppressed	71	18	140	7	0.22	0.16–0.30	< 0.001
People living with HIV immunosuppression unknown	100	26	144	7	0.17	0.12–0.23	< 0.001
HIV status unknown	4	1	152	7	4.34	1.58–11.93	0.004
**HIV-positive if unknown immunosuppression status imputed as people living with HIV not immunosuppressed**							
People not living with HIV	156	40	1442	69	**Reference**	**Reference**	-
People living with HIV not immunosuppressed	156	40	357	17	0.26	0.20–0.34	< 0.001
People living with HIV immunosuppressed	71	18	140	7	0.22	0.16–0.30	< 0.001
HIV status unknown	4	1	152	7	4.61	1.67–12.69	0.003
**HIV-positive if unknown immunosuppression status imputed as people living with HIV immunosuppressed**							
People not living with HIV	156	40	1442	69	**Reference**	**Reference**	-
People living with HIV not immunosuppressed	56	14	268	13	0.53	0.38–0.74	< 0.001
People living with HIV immunosuppressed	171	44	229	11	0.15	0.11–0.19	< 0.001
HIV status unknown	4	1	152	7	4.29	1.56–11.80	0.005

OR, odds ratio; CI, confidence interval; HIV, human immunodeficiency virus.

## Discussion

In this study, we compared 387 influenza-infected individuals in 2016 through 2019 to 2091 SARS-CoV-2 infected individuals in 2020 through 2021 within the pneumonia surveillance programme. Individuals hospitalised with influenza were more likely to be living with HIV (irrespective of immunosuppression status), have asthma, and be underweight. Individuals with SARS-CoV-2 infection early in the pandemic were more likely to be overweight or obese, have diabetes and die in hospital rather than those with influenza.

Previously published data from our pneumonia surveillance programme in South Africa showed that HIV and severe HIV immunosuppression were risk factors for severe influenza.^5,26^ In the United States (US), there was an increased risk of influenza-associated mortality among people living with HIV, even after introduction of antiretroviral treatment.^27^ Additionally, HIV has also been identified as a risk factor for severe disease and death among those with SARS-CoV-2 infection.^4^ However, in our study, when comparing early pandemic SARS-CoV-2 infection with influenza infection, hospitalised individuals with SARS-CoV-2 were less likely to be living with HIV (immunosuppressed and not immunosuppressed). Although HIV is a risk factor for severe influenza^28^ and COVID-19, we showed that the risk of severe disease in people living with HIV is higher among those with influenza rather than SARS-CoV-2. In this study, SARS-CoV-2 was a novel virus and individuals had no immunity for SARS-CoV-2 infection, in contrast to seasonal influenza where most adults would have had multiple previous exposures to influenza.^29^ This likely contributed to increased levels of vulnerability to severe COVID-19 even in individuals with no underlying illness. In contrast, there have been a few decades of exposure to the influenza virus, and some immunity has been formed in the general popualtion, which may reduce disease severity^30^, especially when compared to individuals with underlying conditions, including people living with HIV.

Influenza-infected individuals were more likely to be asthmatic rather than SARS-CoV-2 infected individuals. It is hypothesised that inhaler-mediated corticosteroid use may decrease angiotensin-converting enzyme (ACE2) expression, thus decreasing SARS-CoV-2 cell-entry efficiency.^31^ This could potentially explain why those hospitalised with SARS-CoV-2 infection were less likely to have asthma, although we did not collect information on corticosteroid use. Additionally, being an underweight adult is also a risk factor for severe influenza.^32^ Malnutrition in particular has been associated with severe influenza in children,^5^ but data on the effects of being underweight in adults are limited.^33^ In our study, those hospitalised with influenza were more likely to be underweight.

Non-communicable diseases such as diabetes and obesity are emerging epidemics, and together with infectious diseases, add an additional burden on already resource-limited healthcare systems in South Africa.^34^ Individuals hospitalised with SARS-CoV-2 early in the pandemic were more likely to be overweight, or obese compared to patients with influenza. Studies conducted in China^35^ identified obesity as a risk factor for severe COVID-19 and several reviews have confirmed this.^36,37,38^ Obesity increases ACE2 expression, which facilitates the entry of SARS-CoV-2 into host cells. Obesity is also linked to reduced immune function and increases inflammation which may affect the lung parenchyma and bronchi.^39^

Similarly, compared to influenza, patients hospitalised with SARS-CoV-2 early in the pandemic were more likely to have diabetes. In a study in France, individuals with diabetes were admitted to hospital for COVID-19 (01 March – 30 April 2020) than influenza (01 December 2018 – February 2019).^12^ In South Africa, elevated risk of COVID-19 death was associated with poor glycaemic control diabetes.^40^ In diabetic patients, the exaggerated immune response to SARS-CoV-2 infection, including inflammation, endothelial dysfunction, and oxidative stress, contributes to a more severe disease episode.^41^ The response in increased vascular permeability, pulmonary thrombosis and/or cytokine storm in individuals with diabetes and SARS-CoV-2 infection, all likely contribute to increased severity.^41^ Individuals with seasonal influenza typically do not have a cytokine storm, possibly explaining why the magnitude of risk in diabetic patients is higher for SARS-CoV-2 than influenza.^42^

We observed higher odds of in-hospital mortality for SARS-CoV-2 infected patients compared to influenza infected patients. Almost thrice as many patients with SARS-CoV-2 infection died, compared to influenza (17% vs 6%). Higher mortality rates for SARS-CoV-2 compared to influenza are well described, as in a French study comparing individuals hospitalised with COVID-19 to seasonal influenza^12^ and with another study in Germany in 2020 where the mortality rate was 14% for individuals with SARS-CoV-2 infection and 6% for influenza.^17^ In a study with patients admitted to a tertiary care centre in Boston (US), the mortality rate was 20% for SARS-CoV-2 and 3% for influenza.^43^ The in-hospital mortality rate for the 2009 H1N1 influenza pandemic in the USA was estimated at 2.9%.^44^ The higher in-hospital mortality rate for SARS-CoV-2 is likely a result of SARS-CoV-2 being a novel pathogen in a naïve population, with no prior immunity either because of previous infection or vaccination compared to influenza following decades of exposure.^45^ The severity of SARS-CoV-2 might decline as the population gains immunity through natural infection and/or vaccination as well as the intrinsic lower severity of the Omicron variant, compared to the original variant and other variants of concern. This association of SARS-CoV-2 and mortality may change however, when population immunity to SARS-CoV-2 is higher and similar to that of influenza. Moreover, in-hospital mortality for those with SARS-CoV-2 was higher when hospitals were at higher capacity,^46^ which may have influenced the outcome of a higher in-hospital mortality of those with SARS-CoV-2 infection.

Our study had a number of limitations. Because of the small number of influenza cases detected during 2020–2021 SARS-CoV-2 pandemic, we could not compare the patients enrolled in the same time period. We therefore compared four seasons of influenza infections to four waves of SARS-CoV-2 infections. Additionally, deaths may have been underestimated, as more severe patients were less likely able to consent to enrolment in the study, but this bias was likely similar during the influenza and COVID-19 periods.^10^ A higher proportion of individuals tested for influenza did not have data on immunosuppression in the 2016–2019 period. However, influenza hospitalisation was high, irrespective of immunosuppression status. This could have possibly been further explored with antiretroviral data, which was unavailable. Health seeking behaviour could have changed between 2016–2019 and 2020–2021 period, as some people living with HIV might have been wary of getting medication or care in a facility during the COVID-19 pandemic. On sensitivity analysis (Table 3), when classifying all people living with HIV unknown immunosuppression as either immunosuppressed or non-immune supressed, individuals hospitalised with influenza were still more likely to be people living with HIV immunosuppressed or non-immunosuppressed, compared to those with SARS-CoV-2 infection. We could also not examine differences in clinical presentation, because of the change in the surveillance case definition to incorporate suspected COVID-19 cases in March 2020. With the change in case definition between 2016–2019 and 2020–2021, there was also a limitation in comparing the two groups, as there may have been non-respiratory cases enrolled in the 2020–2021 period, whereas the 2016–2019 group only included respiratory disease cases. The two different laboratory testing methods were also considered a limitation, but it is assumed that the difference in sensitivity and specificity of these tests are negligible.^47,48^ Finally, this study was not powered to assess the risk of comorbidities individually such as chronic renal and liver disease, pregnancy, hypertension or neurological disorders, because of the low prevalence of these conditions in our study population. Furthermore, comparative studies of characteristics of influenza and SARS-CoV-2 hospitalised patients are needed as SARS-CoV-2 becomes an endemic pathogen and many more people gain natural immunity to the pathogen post the initial pandemic years.

## Conclusion

In conclusion, we observed a higher in-hospital mortality rate for SARS-CoV-2 infected individuals in the first 2 years of the pandemic compared to seasonal influenza prior to the pandemic. The SARS-CoV-2 infected hospitalised individuals were more likely to be overweight, obese or have diabetes, and less likely to be living with HIV, underweight or asthmatic, than influenza-infected hospitalised individuals. Identifying groups at high risk of severe disease could help to better monitor, prevent, and control SARS-CoV-2 or influenza severe disease when these two pathogens co-circulate. Differences identified between hospitalised patients with influenza or SARS-CoV-2 infection may change as SARS-CoV-2 becomes endemic, and population immunity is higher. Ongoing systematic surveillance should continue in order to be able to detect any changes in the epidemiology and severity of influenza and SARS-CoV-2 infections over time.
